# Biotechnological Potential of Agro Residues for Economical Production of Thermoalkali-Stable Pectinase by *Bacillus pumilus* dcsr1 by Solid-State Fermentation and Its Efficacy in the Treatment of Ramie Fibres

**DOI:** 10.1155/2012/281384

**Published:** 2012-08-08

**Authors:** Deepak Chand Sharma, T. Satyanarayana

**Affiliations:** ^1^Department of Microbiology, Chaudhary Charan Singh University, Meerut 250 004, India; ^2^Department of Microbiology, University of Delhi South Campus, Benito Juarez Road, New Delhi 110 021, India

## Abstract

The production of a thermostable and highly alkaline pectinase by *Bacillus pumilus* dcsr1 was optimized in solid-state fermentation (SSF) and the impact of various treatments (chemical, enzymatic, and in combination) on the quality of ramie fibres was investigated. Maximum enzyme titer (348.0 ± 11.8 Ug^−1^ DBB) in SSF was attained, when a mixture of agro-residues (sesame oilseed cake, wheat bran, and citrus pectin, 1 : 1 : 0.01) was moistened with mineral salt solution (*a*_*w*_ 0.92, pH 9.0) at a substrate-to-moistening agent ratio of 1 : 2.5 and inoculated with 25% of 24 h old inoculum, in 144 h at 40°C. Parametric optimization in SSF resulted in 1.7-fold enhancement in the enzyme production as compared to that recorded in unoptimized conditions. A 14.2-fold higher enzyme production was attained in SSF as compared to that in submerged fermentation (SmF). The treatment with the enzyme significantly improved tensile strength and Young's modulus, reduction in brittleness, redness and yellowness, and increase in the strength and brightness of ramie fibres.

## 1. Introduction

Solid-state fermentation (SSF) takes place in absence or near absence of free flowing water [1] and is of special economic interest for countries having abundant biomass and agro-industrial residues. The solid substrates act as source of carbon, nitrogen, minerals, and growth factors and have the capacity to absorb water in order to meet the growth requirements of microbes. The possibility of using SSF for pectinase production has been shown using different agro-industrial residues such as wheat bran [2–4], apple pomace [5, 6], lemon and orange peel [7–9], sugar cane bagasse [10], tomato pomace [11], and sugar beet pulp [12]. Very few detailed investigations have, however, been conducted on the production of alkaline and thermostable pectinases in SSF using bacterial strains [2, 13–16].

Pectinases optimally active at acidic pH find extensive applications in the extraction, clarification, and liquefaction of fruit juices and wines [17, 18], while alkaline pectinases find applications in textile industry for retting of plant fibres, manufacturing of cotton fabrics, and enzymatic polishing of jute/cotton-blended fabrics, in paper industry to solve the retention problems in mechanical pulp bleaching, in the treatment of pulp and paper mill effluents, and for improving the quality of black tea [15, 19–24]. Ramie (china grass) fibre is considered one of the longest, strongest, and silkiest plant fibres known [25]. The cellulose fibres of ramie are also arranged in bundles parallel to the longitudinal axis of the stem and are embedded in a pectic polysaccharide network as present in bast fibre plants [26]. The gummy material present on the surface of fibre should be removed without causing damage to the structure of fibre to maintain its flexibility. Development of high brittleness is a major disadvantage of the fibre for the application in textile industry [25], and the condition is caused by the nonspecific treatment with chemicals or with crude enzyme preparations having cellulase. Hence, in this investigation an attempt has been made to optimize various physical and chemical parameters to produce elevated levels of thermo-alkali stable pectinase using agriculture residues in SSF by the alkali tolerant *Bacillus pumilus *dcsr1 (GenBank accession AY426610). The-cost effective production of this valuable enzyme for the processing of ramie fibres [15] will enhance its applicability in the development of green technology. The effect of various treatments on the physical properties of ramie fibre is also discussed. 

## 2. Materials and Methods

### 2.1. Source of the Strain and Its Identification

The bacterial strain was isolated from a soil sample collected from Rohtak, Haryana (India), maintained on nutrient agar slants at 4°C, and also stored as glycerol stocks at −20°C. The strain was identified as *B. pumilus *dcsr1 (99% homology) based on 16S rDNA sequence analysis (GenBank accession number AY426610).

### 2.2. Pretreatment of Solid Substrates

Agro-residues used in this investigation were collected from local market and washed with water (to remove the dirt and soluble impurities), air-dried, and ground to uniform size.

### 2.3. Production of Alkaline Pectinase in Wheat Bran

The fermentation was carried out in 250 mL Erlenmeyer flasks containing 5 g of pretreated wheat bran moistened with 12.5 mL of distilled water and autoclaved at 121°C (15 Lb psi) for 15 min. The flasks were cooled and inoculated with 20% (w/v) of the bacterial culture (CFU *≈* 1.58 × 10^8^ mL^−1^) cultivated for 24 h in lactose-pectin-yeast extract broth [gL^−1^: lactose 1.90, citrus pectin 3.50, yeast extract 1.00, casein 1.8, K_2_HPO_4_ 1.00, MgSO_4_·7H_2_O 2.50, Na_2_HPO_4_ 5.00, 2 mL micronutrient solution, and pH 8.0 [15]]. The inoculum was mixed thoroughly by gently tapping the bottom of the flasks and incubated in a humidified chamber (YORCO Pvt. Ltd., New Delhi, India) maintained at 80% relative humidity (RH) and 40°C for 96 h. Periodically the contents of the flasks were mixed by gentle tapping against the palm. 

### 2.4. Observation of Bacterial Growth on Wheat Bran

Forty-eight-hour-old bacterial growth on wheat bran was fixed in glutaraldehyde (2.5%, for 4 h) and then washed with phosphate buffer. The fixed samples were dehydrated in ascending grades of alcohol (30%, 50%, 70%, 80%, 90%, and 100%) for 30 min, dried in 1,1,1,3,3,3-hexamethyldisilazane (HMDS) and gold film (thickness 20–25 nm) was created with the help of agar sputter coater. The bacterial growth was examined under scanning electron microscope (Leo 435 UP, Cambridge, UK).

### 2.5. Extraction of the Enzyme from the Fermented Substrate

After 96 h, contents of the flasks were extracted twice with 25 mL of buffer (0.01 M sodium phosphate buffer, pH 7.0) by constant shaking for 1 h in a rotary shaker at 200 rpm. The extract was squeezed through muslin cloth and centrifuged at 10,000 rpm for 20 min at 4°C. The cell-free supernatant thus obtained was used as the source of extracellular alkaline pectinase. The fermented substrate was dried to constant weight at 80°C for determining dry weight of the bacterial bran. The enzyme titre is expressed as units gram^−1^ dry bacterial bran (Ug^−1^ DBB). 

### 2.6. Alkaline Pectinase Assay

Alkaline pectinase in the cell-free culture filtrate was assayed according to Sharma and Satyanarayana [15]. One unit of pectinase is defined as the amount of enzyme that liberates 1 *μ*mole reducing sugars as D-galacturonic acid min^−1^ under the assay conditions.

### 2.7. Effect of Various Agro Residues on Alkaline Pectinase Production

The bacterium was grown in Erlenmeyer flasks (250 mL) containing 5 g of different pre-treated agro-residues (citrus peel, pomegranate peel, citrus peel powder, pineapple pulp, spent tea leaves, sunflower leaf, cotton oilseed cake, mustard oilseed cake, sesame oilseed cake, wheat straw, wheat bran, sun hemp stalks, sunflower stalks, sunflower, sugarcane bagasse, ramie fibre, sun hemp fibre and rice straw). The bacterial strain was also cultivated in flasks containing different quantities (2.5, 5, 10, 15, 20 g) and combinations of solid substrates (wheat bran, sesame oilseed cake, mustard oilseed cake, and citrus peel).

### 2.8. Effect of Various Moistening Agents on Alkaline Pectinase Production

For selecting the best moistening agent, mixed solid substrate (wheat bran, sesame oilseed cake and citrus pectin in 1 : 1 : 0.5 ratio) was moistened with different salt solutions (SS) (gL^−1^: SS1. (NH_4_)_2_SO_4_ 4.00, KH_2_PO_4_ 10.00, CaCl_2_ 0.30, FeSO_4_ 0.30, MgSO_4_ 0.30; SS2. (NH_4_)_2_SO_4_ 0.40, KH_2_PO_4_ 2.10, CaCl_2_ 0.30, FeSO_4_ 0.11, MnSO_4_ 0.30; SS3. CaCl_2_ 0.10, MgSO_4_·7H_2_O 0.50, FeSO_4_ 0.10; SS4. NH_4_NO_3_ 2.00, K_2_HPO_4_ 6.00, KCl 0.50, MgSO_4_ 0.50; SS5. K_2_HPO_4_ 0.10, (NH_4_)_2_H_2_PO_4_ 1.00, MgSO_4_ 0.50, CaCl_2_ 0.10, FeSO_4_ 0.10, MnSO_4_ 0.10; SS6. Na_2_HPO_4_ 11.00, NaH_2_PO_4_ 6.00, KCl 3.00, MgSO_4_ 0.10; SS7. KH_2_PO_4_ 2.40, MgSO_4_·7H_2_O 0.50, CaCl_2_·2H_2_O 0.10), SS8. distilled water, SS9. phosphate buffer, and SS10. tap water were used (12.5 mL/5 g of solid substrate). The pH of the salt solutions was adjusted to 8.0 using 1 N HCl/NaOH.

### 2.9. Effect of Various Physical Parameters on Alkaline Pectinase Production

The ratio of the solid substrate (g) to moistening agent (mL) was varied (1 : 1, 1 : 2, 1 : 2.5, 1 : 3, 1 : 4, and 1 : 5). The inoculum age was optimized by inoculating the substrate with bacterial inoculum grown for 12, 24, 36, 48 25, 60, 72, and 84 h, while the optimum inoculum size was determined by inoculating the substrate with the desired volume of the bacterial culture. To study the effect of pH on enzyme production, the bacterial strain was cultivated on the substrate moistened with salt solutions of varied pH (0.1 M citrate buffer for pH 4 & 5.0; 0.1 M phosphate buffer for pH 6.0 to 8.0; 0.1 M glycine-NaOH buffer for pH 9.0; CAPS buffer (N-cyclohexyl-3-aminopropanesulfonic acid) for pH 10, 10.5 & 11. The effect of temperature was assessed by incubating inoculated flasks at different temperatures (30, 35, 40, 45, and 50°C).

### 2.10. Effect of Water Activity (*a*_*w*_) on Alkaline Pectinase Production

*B. pumilus* was cultivated in flasks containing solid substrate moistened with salt solution 4 with glycerol for attaining different *a*_*w*_ values according to Grajek and Gervais [27].

### 2.11. Treatment of Ramie Fibre and Evaluating the Properties of the Treated Fibres

The ramie fibres were treated according to Sharma and Satyanarayana [15], and the physical properties of the fibres were assessed at National Physical Laboratory (NPL, Delhi, India). Tensile strength, strain, and Young's Modulus were determined by universal testing machine (Instron, USA). The color coordinates of the sample were measured by gonio spectrophotometer (Zeiss, Germany). The source used for illumination was a pulsed xenon lamp having a spectral distribution of a D_65_ illuminant. The measurements were made using a 10° standard observer in the spectral range 300–720 nm. The CIELAB color parameters L* (brightness), a* (redness), and b* (yellowness) were also measured.

## 3. Results and Discussion

**Figure 1:**
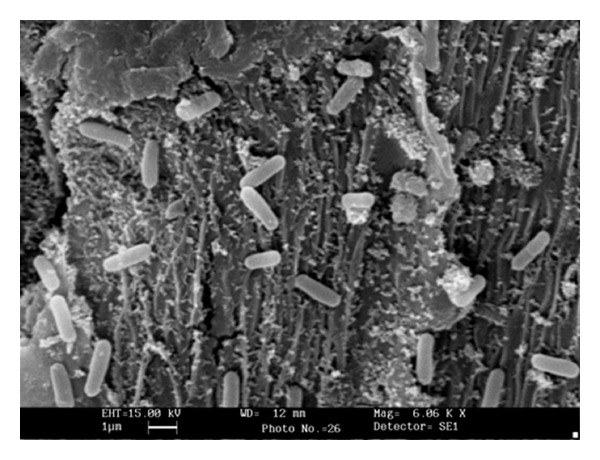
Scanning electron micrograph of fermented solid substrate (wheat bran) showing bacterial cells.

Solid-state fermentation (SSF) has great potential for the development of several bioprocesses and products because of enhanced productivity and prospects of using a wide range of agro-industrial residues as substrates [28, 29]. Efforts have been made to exploit filamentous fungi in SSF for the production of various products, and further attempts are being made to explore the possibility of using bacterial strains in SSF systems [1, 30]. The direct observation of the microbe in solid substrate remains a difficulty in all studies. Scanning electron microscopy (SEM) of the fermented solid substrate revealed luxuriant bacterial growth on wheat bran particles that confirmed amenability of *B. pumilus* dcsr1 to SSF (Figure 1) like some other *Bacillus* spp. [31]. Among different agro-residues tested (Table 1), sesame oilseed cake supported high pectinase production by *B. pumilus* (210.22 ± 8.08 Ug^−1^ DBB). This may be due to the fact that along with pectinase the bacterial strain also produced lipase and xylanase (data not presented here), which may help in efficient utilization of these solid substrates. A high enzyme titre (232.12 ± 12.12 Ug^−1^ DBB) was attained when wheat bran was mixed with sesame oil seed cake and citrus pectin (1 : 1 : 0.01 ratio) as compared to other combinations (Table 1). Wheat bran is known to be a good solid substrate [32], supplementation of this with other solid substrates led to increase in enzyme yield [17], the enhancement in amylase production was also recorded on supplementing wheat bran with oil seed cakes [28]. Although wheat bran provides good support and availability of water and oxygen for the bacterium, a supplementation with additional carbon and nitrogen source increases the “carrying capacity” of the wheat bran. The bacterium produced high titres of enzyme when granules of citrus peel were used instead of citrus peel powder that may be attributed to the fact that in SSF the solid substrate not only supplies the necessary nutrients to the microorganism but also provides anchorage on the increased surface area with better aeration. Therefore, the particle size and the chemical composition of the substrate are very important [33, 34]. The low enzyme titre in wheat straw (23.17 ± 2.23 Ug^−1^ DBB), sugar cane bagasse (27.65 ± 1.02 Ug^−1^ DBB), rice husk (45.09 ± 5.60 Ug^−1^ DBB), sun hemp stalk (34.75 ± 1.55 Ug^−1^ DBB), and sunflower leaf (32.51 ± 0.23 Ug^−1^ DBB) might be due to their high lignin and silica contents [34].

Quantity of solid substrate taken in a container is an important parameter that needs to be optimized, since higher amount of substrate interrupts heat transfer and aeration. A high enzyme titre (219.05 ± 11.73 Ug^−1^ DBB) was attained when 5/10 g of substrate were taken in Erlenmeyer flasks of 250 mL, as observed for the production of xylanase by *A. niger* [29]. The elevated level of substrate may hinder the optimal transfer of heat and oxygen, and thus, lead to low enzyme titre.

**Figure 2:**
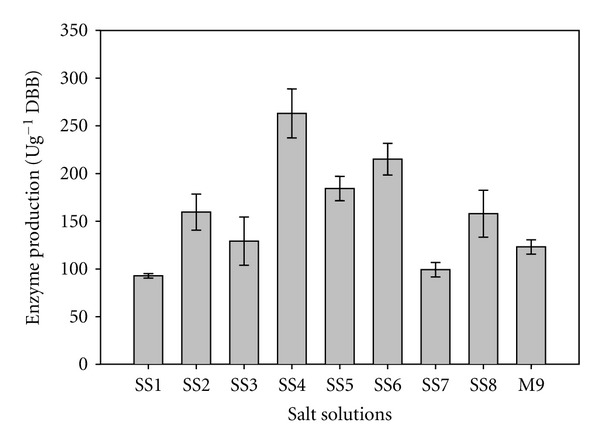
Effect of various salt solutions on alkaline pectinase production in SSF.

Salt solutions not only provide moisture to the solid substrate but also provide additional nutrients and cations to the bacterium. Various moistening agents such as tap water [35], phosphate buffer [36, 37], and salt solutions [38] have been used in SSF. A marked variation in thermo-alkali-stable pectinase production by *B. pumilus* dcsr1 was observed when different salt solutions were used to moisten solid substrate (Figure 2). The enzyme yield was higher in salt solution 4 (263 ± 25.7 Ug^−1^ DBB) containing ammonium nitrate as one of its constituents than others, which might have provided an additional nitrogen source for supporting bacterial growth.

**Figure 3:**
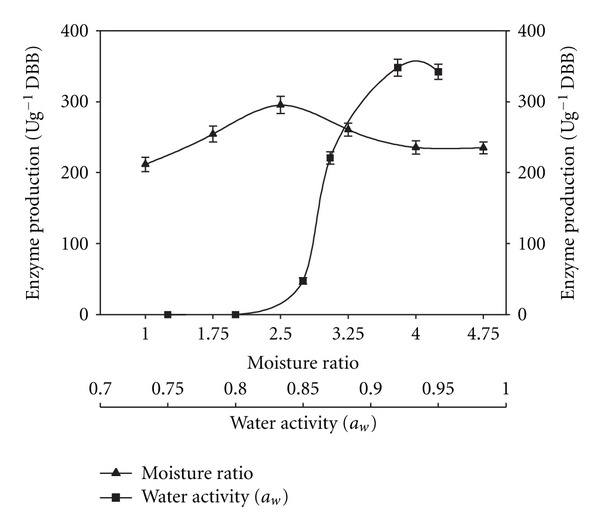
Effect of moisture level and water activity on alkaline pectinase production in SSF.

Among all culture variables, the initial moisture level is one of the most critical parameters in SSF because it determines the swelling, water tension (water surface tension), and solubility and availability of nutrients to the microbe for their growth. Moisture content of the fermentation medium determines the success of the process [29, 33]. The alkaline pectinase titre was (295 ± 12.2 Ug^−1^ DBB) higher when the substrate to moistening agent ratio was 1 : 2.5 (w/v) (Figure 3). Lower enzyme titres below this ratio could be due to the reduced solubility of nutrients present in the solid substrate, a lower degree of swelling, and high water tension [39]. The production of *α*-amylase by *B. coagulans* [28] and xylanase by *B. licheniformis* [40] was also high at this moisture level. A far lower moisture ratio (1 : 1) was reported for xylanase production by *Bacillus* sp. A-009 [41]. The high moisture level may result in decreased porosity, changed particle structure, increased stickiness, reduced gas volume, and decreased diffusion that resulted in lower oxygen transfer and less growth.

**Figure 4:**
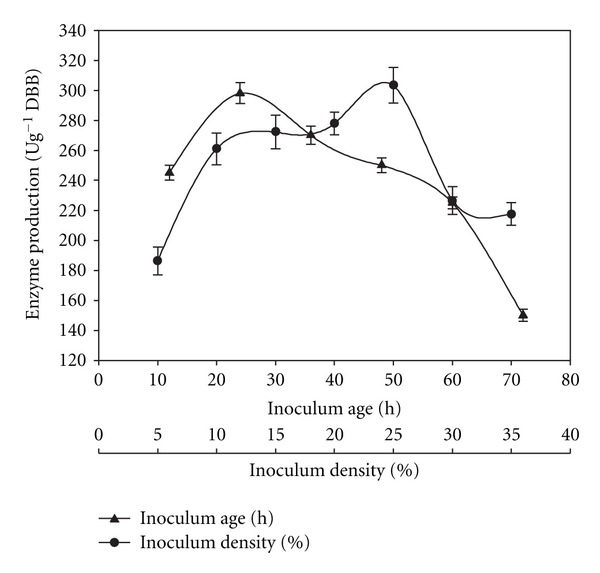
Effect of inoculum age and density on alkaline pectinase production in SSF.

The inoculum age and size directly affect microbial growth and enzyme production. An inoculum size of 25% of a 24-hour-old bacterial culture supported an enzyme titre of 303 ± 12 Ug^−1^ DBB (Figure 4). At higher inoculum levels, the production declined; this could be due to competition among bacterial cells for nutrients. The younger cultures of *B. pumilus*, being nonsporulating, entered the growth phase very soon, while the cultures older than 36 h produced spores which took longer time to germinate, and therefore, the enzyme production was low as previously reported [42].

**Figure 5:**
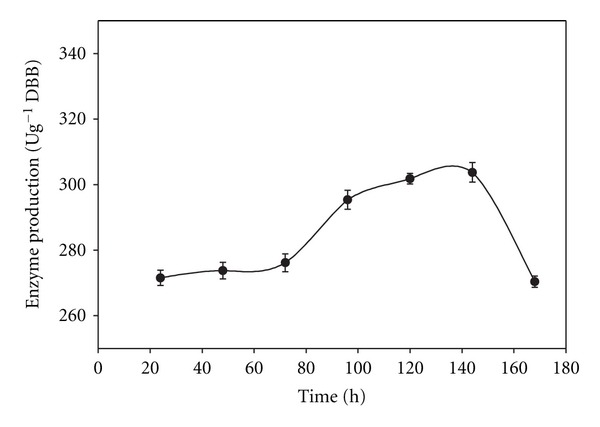
Alkaline pectinase production in SSF at various incubation periods.

The enzyme secretion by *B. pumilus *reached a peak in 120 h and remained constant till 144 h, followed by a decline (Figure 5). The incubation time is the characteristics of the strain, depends on growth rate in substrate and enzyme production pattern [34]. The incubation time for SSF ranged between 30 and 144 h for different microbial strains [36, 37].

**Figure 6:**
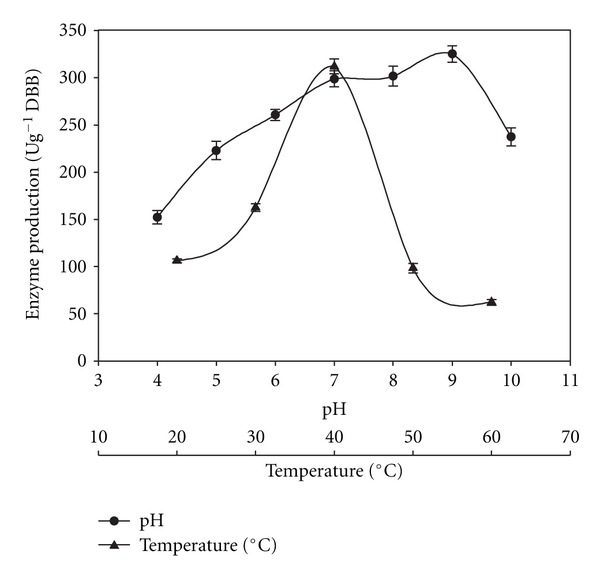
Effect of pH and temperature on enzyme production.

Pectinase titre increased when pH of solid substrate was increased from 4 to 9, and thereafter, it declined (Figure 6). A maximum enzyme production was recorded at pH 9 and 40°C (Figure 6). The selection of pH and temperature in SSF is based on the optimum pH and temperature for growth of microbes. The pH is known to act synergistically with other environmental parameters besides being a regulatory parameter in biotechnological processes [34].

Water activity (*a*_*w*_) indicates the amount of unbound water available in the surroundings of the microorganism. It is related to the water content of the substrate, although it is not equal to moisture content. The water activity (*a*_*w*_) of the substrate is important in SSF because at relatively low water availability, growth and metabolism can be limited [43]. The extrapolation of *a*_*w*_ could be useful in modifying metabolite production or secretion of a product [43, 44]. The production of alkaline pectinase by *B. pumilus* followed the same trend (Figure 3). The ability of bacterium to grow on solid substrate at low water activity appeared to be an adaptation of the bacterium for survival in the soil.

Parametric optimization under SSF resulted in overall 1.7-fold increase in enzyme production, while a 14.2-fold enhancement was achieved in SSF as compared to statistically optimized medium in SmF [15]. There are several reports claiming that the SSF technique yields higher enzyme titres than SmF [29, 40, 45–47], but many of them have not compared alkaline pectinase production in both processes [4, 48]. Enhancement in enzyme production in SSF as compared to that in SmF could be due to the intimate contact of the organism to the substrate and minimization of catabolite repression [17]. 

Ramie fibres were treated with the alkaline pectinase produced by *B. pumilus *dcsr1 [15] and changes in the physical properties of fibre were evaluated. The comparison of physical properties of fibre after various treatments showed significant variations (Table 2). The tensile strength and Young's Modulus of the fibre increased after combined treatment of fibres with NaOH (0.04%) followed by the enzyme (300 U/g dry fibre), which resulted in the reduction of brittleness, redness, yellowness, and increase in the strength and brightness of the fibre. An increase in Young's Modulus in NaOH-(12%) treated fibre could be due to the deeper penetration of the enzyme and chemicals into the fibre and digestion of other polymers present on the surface. This nonspecific digestion definitely caused slight increase in elasticity but led to a compromise in tensile strength of the fibre. Nonspecific digestion with chemicals leads to high brightness due to the digestion of other polymers including cellulose, which was evident from the decrease in strength of the fibre. 

## 4. Conclusions

The amenability of *Bacillus pumilus *dcsr1 to solid state fermentation and its ability to produce thermo-alkali-stable pectinase using cost-effective agro-residue were successfully established. A 14.2 fold enhancement in enzyme production was achieved in SSF in comparison with that in submerged fermentation. The enzyme has been found to be useful in the environment-friendly processing of ramie fibres without compromising the quality of treated fibres.

## Figures and Tables

**Table 1:** Alkaline pectinase production on various agro-residues alone and their combinations.

S. no.	Solid substrates used	Enzyme production (Ug^−1^ DBB)
(1)	Wheat straw	23.17 ± 2.23
(2)	Rice husk	45.09 ± 5.60
(3)	Wheat bran	160.97 ± 3.11
(4)	Sesame oil seed cake	210.22 ± 8.08
(5)	Mustard oil seed cake	204.99 ± 4.22
(6)	Cotton oil seed cake	37.09 ± 7.07
(7)	Sugarcane bagasse	27.65 ± 1.02
(8)	Pomegranate peel	72.03 ± 9.58
(9)	Pineapple pulp	140.16 ± 5.23
(10)	Sun hemp fibre	73.18 ± 6.48
(11)	Sun hemp stalk	34.75 ± 1.55
(12)	Sunflower seed	89.24 ± 5.88
(13)	Sunflower stalk	110.34 ± 0.93
(14)	Sunflower leaf	32.51 ± 0.23
(15)	Citrus peel	162.22 ± 4.28
(16)	Citrus peel powder	41.25 ± 0.92
(17)	Citrus fruit pulp	60.66 ± 2.22
(18)	Spent tea leaves	69.99 ± 0.51
(19)	Ramie fibres	116.18 ± 6.14
(20)	Wheat bran + sesame oil seed cake (1 : 1)	217.18 ± 7.18
(21)	Wheat bran + sesame oil seed cake + Citrus pectin (1 : 1 : 0.01)	232.12 ± 12.12
(22)	Wheat bran + citrus peel (1 : 1)	140.23 ± 7.23
(23)	Wheat bran + citrus peel + Sesame oil seed cake (1 : 1 : 1)	59.52 ± 0.95
(24)	Wheat bran + mustard oil seed cake (1 : 1)	195.55 ± 1.55

**Table 2:** Effect of various treatments on the physical properties of ramie fibre.

Type of treatment given to the fibre	Stress at peak (MPa)	Young's modulus (GPa)	Strain at max. load (%)	Brightness (*l*)	Redness (*a*)	Yellowness (*b*)
Raw fibre (Control)	488.94	23.40	8.571	108.25	1.48	8.55
Chemical (12% NaOH)	478.73	26.06	6.78	121	0.91	3.42
Commercial fibre	374.1	13.69	13.21	115	1.03	4.75
Enzyme + NaOH (0.04%)	848.6	25.89	9.93	118.87	0.98	4.02
